# Basilar artery vasospasm after pretuncal non-aneurysmal subarachnoid hemorrhage responding to milrinone

**DOI:** 10.17712/nsj.2017.2.20160451

**Published:** 2017-04

**Authors:** Abdulrahman Y. Alturki, Abdullah S. Alamri, Mohamed M. Badawy, Benjamin W. Lo

**Affiliations:** *From the Neurocritical Care Unit (Alturki, Alamri, Badawy, Lo), Department of Anesthesia (Badawy), Montreal Neurological Hospital, Canada, from the Division of Neurocritical Care (Alturki), Department of Neurosurgery, National Neurosciences Institute, King Fahad Medical City, Riyadh, and from the Department of Neurology (Alamri), King Fahd hospital, University of Dammam, Department of Neurology (Alamri), Imam Abdulrahman Bin Faisal University, Dammam, Kingdom of Saudi Arabia*

## Abstract

Pretruncal (perimesencephalic) non-aneurysmal subarachnoid hemorrhage (PNSAH) is uniformly associated with an excellent outcome. Although cerebral vasospasm remains a common complication of SAH and constitutes an important predictor of outcome, in the setting of PNSAH, it is extremely rare. Preturnal non-aneurysmal subarac refers to a subset of SAH patients with a characteristic pattern of localized blood on CT of the head, normal cerebral angiography, and benign course when compared to the aneurysmal SAH population. The presence of radiological or even clinical vasospasm does not exclude the diagnosis of PNSAH. To our knowledge, this is the first case of symptomatic cerebral vasospasm due to PNSAH that responded to milrinone.

Pretruncal (perimesencephalic) non-aneurysmal subarachnoid hemorrhage (PNSAH) is uniformly associated with an excellent outcome. These patients usually present with minimal complaints and require a shorter period of monitoring compared to those with aneurysmal hemorrhage. Another recognized feature of PNASH is the uncommon occurrence of cerebral vasospasm, unlike aneurysmal SAH in which this complication is an important predictor of outcome.^1^ Due to this, the evaluation and management of vasospasm in PNSAH remains controversial due to the rarity of the condition. Milrinone is a potent selective phosphodiesterase III inhibitor that affects cyclic adenosine monophosphate (cAMP) pathways with both inotropic and vasodilator effects, it is gaining popularity in the management of vasospasm due to aneurysmal SAH with good reported outcomes.^2^ This report describes the case of a patient presented with PNSAH, who developed symptomatic vasospasm of the basilar artery that required therapeutic intervention, including the use of milrinone, with satisfactory outcome.

## Case Report

**Figure 1:**
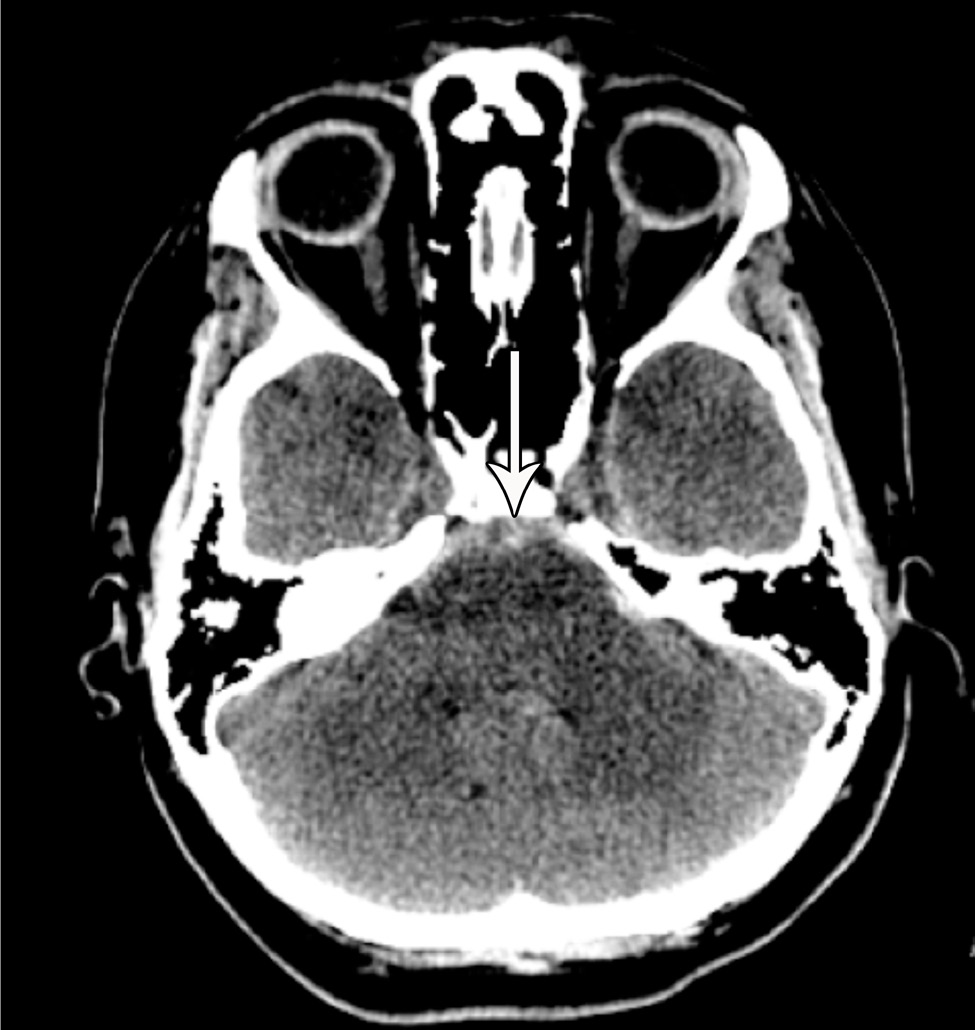
- Head CT demonstrate subarachnoid hemorrhage anterior and lateral to the pons.

**Figure 2:**
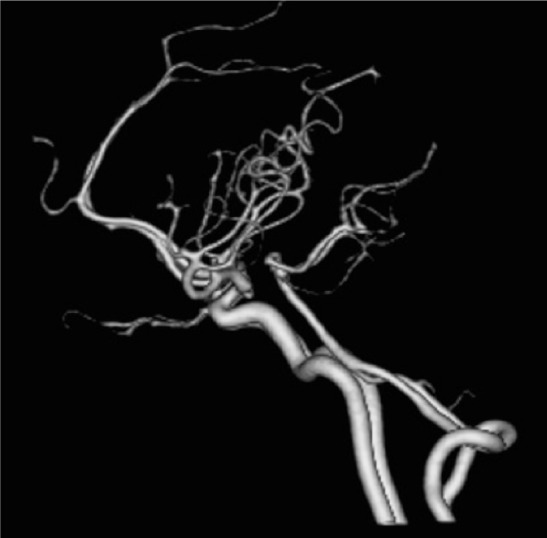
- Lateral view CTA 3D reconstruction done at admission.

**Figure 3:**
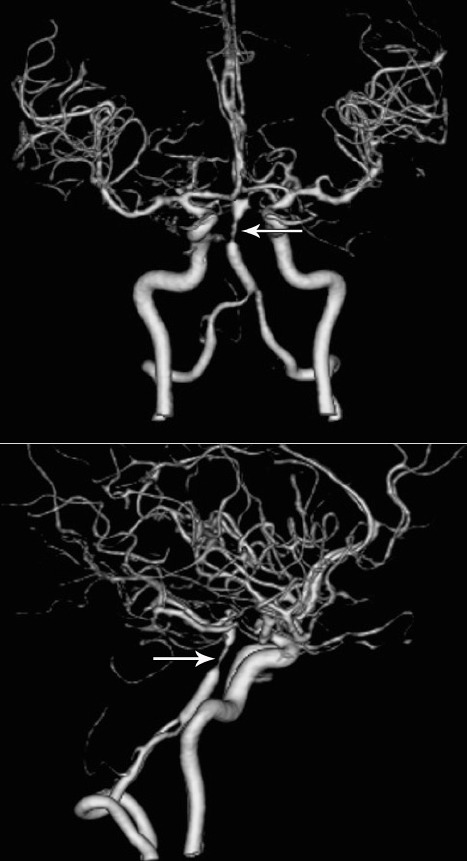
- Anterior-posterior and lateral views CTA 3D reconstruction shows severe upper mid basilar artery vasospasm.

**Figure 4:**
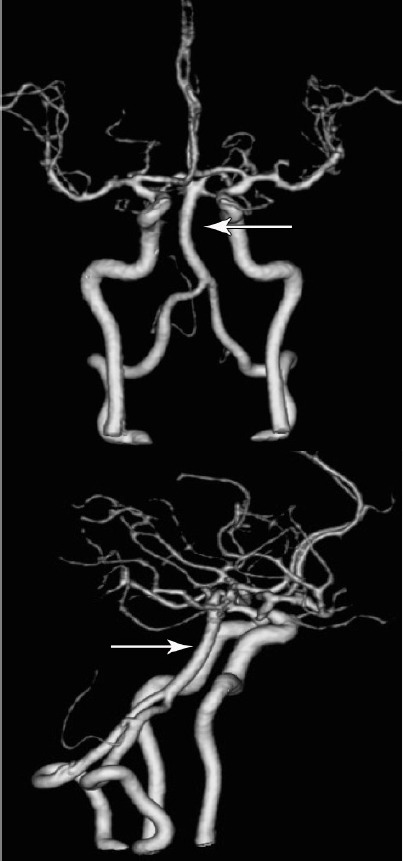
- Anterior-posterior and lateral views CTA 3D reconstruction shows complete resolution of basilar artery vasospasm in response to milrinone therapy.

A 42-year-old female, with a history of two uneventful cesarean sections, who was in her usual state of good health until 2 hours prior to her presentation to the emergency department when as she experienced an acute, sudden, severe headache, manly occipital, associated with neck pain and nausea. Neurological examination was normal on presentation to the hospital. A head computed tomography (CT) (**Figure 1**) demonstrated subarachnoid hemorrhage anterior and lateral to the pons. A CT angiography (CTA) followed by six-vessel cerebral angiogram did not reveal any abnormality of the intracranial vasculature (**Figure 2**). In addition, systemic workup for autoimmune diseases was negative. She was started on nimodipine therapy (60 mg every 4 hours for 21 days from presentation) and was kept in the intensive care unit (ICU) for monitoring for a week due to severe headaches, nausea and vomiting. Transcranial doppler test (TCD) for follow up was not possible due to lack of cranial window. Eventually she was transferred to the ward as the headaches and nausea subsided. As she was being prepared for discharge from the hospital on day 11 post bleed, she developed severe headache, fatigue, nausea, vomiting and neck pain. Neurological examination revealed neck rigidity and photophobia. computed tomography head did not show a new hemorrhage and a CTA (**Figure 3**) demonstrated severe upper basilar artery vasospasm. At this point, she was transferred to ICU for initiation of the Montreal Neurological Hospital protocol^2^ which includes a milrinone bolus of 5 mg intravenously (IV) followed by 0.5 mcg/kg/min continuous IV infusion. Her symptoms resolved within a few hours of admission and a CTA was repeated 48 hours after milrinone was started (**Figure 4**). Two days after CTA, milrinone infusion was reduced to 0.25 mcg/kg/min for 24 hours, then it was stopped. She was discharged home in a stable condition without neurological deficit.

## Discussion

The reported proportions of cases of non-traumatic and non-aneurysmal subarachnoid hemorrhage that are PNSAH are as high as 68%. On early generation CT scans, the center of such bleed was noted around the midbrain (perimesencephalic). Recently, depending on the pattern of blood distribution in the subarachnoid space on CT scan, non-aneurysmal subarachnoid hemorrhage can be divided into PNSAH if the pattern of blood is located ventral to the brain stem (interpeduncular cistern), or non-PNSAH if the blood distribution involves other basal cisterns (sylvian, interhemispheric, and cerebellopontine cisterns).^3^

In the majority of cases of PNSAH, the etiology is not defined, even after exhaustive evaluation. Due to this, assumptions regarding its origin are proposed, backed up by few case reports, which include: rupture of a local venous/ capillary structure, perforating artery, cryptic vascular malformation, high cervical spinal dural arteriovenous fistula and capillary telangiectasia. The search for an etiology has even included exploratory craniotomies in this patient population and has frequently revealed no visible source for the SAH.

Good outcomes are the rule with most series reporting no poor outcomes. Long-term follow-up studies reveal that life expectancy is not altered and rebleeding is exceptional. Patients had residual complaints consisting of headaches, irritability, depression, forgetfulness, weariness, and diminished endurance.^4^,^5^

When compared to aneurysmal SAH population, patients with PNSAH rarely develop angiographic and much less clinical vasospasm. There is large variation in reporting angiographic vasospasm (3-20%). These differences can be explained by timing and number of cerebral angiograms performed. When angiographic vasospasm is present, it is usually mild and focal, mainly affecting the posterior circulation,^4^,^6^ but severe and diffuse vasospasm has also been reported. When it occurs, clinical vasospasm is usually transient, associated with mild neurological findings and far less common than that seen radiographic imaging. When clinical or radiological vasospasm is seen in the context of PNSAH, it often follows the angiography itself leading some to believe that it may in fact be a complication of the diagnostic procedure itself.^7^

The exceedingly low incidence of vasospasm and better outcomes of patients with PNSAH compared to those of patients with aneurysmal SAH could not be explained by the lower volume of blood in these patients; the reason for this difference has not yet been elucidated. Patients with PNSAH had better clinical outcomes and were much less likely to develop delayed ischemia in angiographically-matched negative and positive patients.

Despite its relatively benign outcome, a cautious approach to the clinical management of patients with PNSAH is advised. Patients should be treated as though there is an underlying aneurysm until this has been satisfactorily excluded because ruptured posterior circulation aneurysms can lead to SAH patterns similar to those seen in PNSAH.^8^

In the extraordinary circumstance where clinical vasospasm develops, similar management approaches to vasospasm secondary to aneurysmal SAH should be considered (elevating blood pressure, etc.), including the use of milrinone.^2^

Cerebrovascular smooth muscles contain large amounts of phosphodiesterase making it a suitable target for milrinone. The latter decreases wall thickness, increases the amount of cAMP, reduces corrugation of the elastic lamina of isolated arteries in animals with chronic vasospasm,^9^ and lastly has anti-inflammatory effects through the inhibition of interleukins.

Milrinone administered by different routs (intravenously, intra-arterially and intracisternally) has been used successfully in the battle against cerebral vasospasm due to aneurysmal SAH.^2^,^10^ This case shows that the drug can also be useful in the context of vasospasm due to PNSAH.

In conclusion, PNSAH refers to a subset of SAH patients with a characteristic pattern of localized blood on CT, normal cerebral angiography, and a benign course when compared to the aneurysmal SAH population. The presence of radiological or even clinical vasospasm does not exclude the diagnosis of PNSAH, with a management that coincides with the aneurysmal SAH literature. To our knowledge, this is the first case of symptomatic cerebral vasospasm due to PNSAH that responded to milrinone.

## References

[1] Hsu W, Pradilla G, Garonzik IM, Conway JE (2010). Pretruncal nonaneurysmal subarachnoid hemorrhage causing basilar artery vasospasm. Neurocritical care.

[2] Lannes M, Teitelbaum J, del Pilar Cortés M, Cardoso M, Angle M (2012). Milrinone and homeostasis to treat cerebral vasospasm associated with subarachnoid hemorrhage:the Montreal Neurological Hospital protocol. Neurocritical care.

[3] Kang DH, Park J, Lee SH, Park SH, Kim YS, Hamm IS (2009). Does non-perimesencephalic type non-aneurysmal subarachnoid hemorrhage have a benign prognosis?. J Clin Neurosci.

[4] İldan F, Tuna M, Erman T, Göçer Aİ, Çetinalp E (2002). Prognosis and prognostic factors in nonaneurysmal perimesencephalic hemorrhage:a follow-up study in 29 patients. Surg Neurol.

[5] Greebe P, Rinkel GJ (2007). Life expectancy after perimesencephalic subarachnoid hemorrhage. Stroke.

[6] Jung JY, Kim YB, Lee JW, Huh SK, Lee KC (2006). Spontaneous subarachnoid haemorrhage with negative initial angiography:a review of 143 cases. J Clin Neurosci.

[7] Mayor S, Erro ME, Zazpe I, Gállego J (2008). [Pontine stroke due to vasospasm secondary to perimesencephalic subarachnoid hemorrhage]. Neurologia.

[8] Alén JF, Lagares A, Lobato RD, Gómez PA, Rivas JJ, Ramos A (2003). Comparison between perimesencephalic nonaneurysmal subarachnoid hemorrhage and subarachnoid hemorrhage caused by posterior circulation aneurysms. J Neurosurg.

[9] Nishiguchi M, Ono S, Iseda K, Manabe H, Hishikawa T, Date I (2010). Effect of vasodilation by milrinone, a phosphodiesterase III inhibitor, on vasospastic arteries after a subarachnoid hemorrhage in vitro and in vivo:effectiveness of cisternal injection of milrinone. Neurosurgery.

[10] Sadamasa N, Yoshida K, Narumi O, Chin M, Yamagata S (2014). Milrinone via lumbar subarachnoid catheter for vasospasm after aneurysmal subarachnoid hemorrhage. Neurocrit Care.

